# The description of a new species of the Neotropical land crab genus *Gecarcinus* Leach, 1814 (Crustacea, Decapoda, Brachyura, Gecarcinidae)

**DOI:** 10.3897/zookeys.435.7271

**Published:** 2014-08-18

**Authors:** Robert Perger, Adam Wall

**Affiliations:** 1 Colección Boliviana de Fauna. Casilla 8706, La Paz, Bolivia; 2 Natural History Museum of Los Angeles County, 900 Exposition Blvd., Los Angeles, CA 90007, USA

**Keywords:** Brachyura, Colombia, Ecuador, Gecarcinidae, *Gecarcinus*, Isthmus of Panama, Pacific, new species

## Abstract

In this contribution a new species of the land crab genus *Gecarcinus* Leach, 1814, from the Neotropical Pacific coast of South America is described and illustrated. In addition to its unique body color, *Gecarcinus nobilii* **sp. n.** is distinguished from congeners by a distinctly wider carapace front and differences in the shape of the infraorbital margin. The new species is not isolated from *Gecarcinus* populations from the Pacific coast of Central America by an insurmountable geographic barrier. Considering the closure of the Panamanian Isthmus as a calibration point for morphological divergence between the trans-isthmian mainland populations of *Gecarcinus*, the virtual lack of morphological differentiation (other than color) between them and the distinctness of *G. nobilii* **sp. n.** suggests that *G. nobilii* **sp. n.** evolved from a common ancestor before the Isthmus closed.

## Introduction

Land crabs of the genera *Gecarcinus* Leach, 1814, *Johngarthia* Türkay, 1970, and *Gecarcoidea* H. Milne Edwards, 1837, play an important ecological role on many tropical islands (see Lindquist et al. 2009 for a review). On some islands their biomass may exceed the total mass of animals reported in tropical rain forests in Costa Rica and the central Amazon (Lindquist et al. 2009) and they may occupy the top of the energy pyramid (Burggren and McMahon 1988). Nevertheless, despite their success on islands and the possibility of dispersal of their marine larvae via ocean currents, most species of these genera are absent from continental mainland habitats (Türkay 1987; see also Perger 2014 for a short review). Only *Johngarthia planata* (Stimpson, 1860) (see Perger 2014) and two populations of the Neotropical genus *Gecarcinus* are successfully established on the continental mainland. The Atlantic population, known as *Gecarcinus lateralis* (Fréminville, 1835), occurs along mainland beaches from Florida to Venezuela (Türkay 1970) and is partially sympatric with *Gecarcinus ruricola* (Linnaeus, 1758), which is restricted to Western Atlantic islands. The distribution of the Pacific population, originally described as *Gecarcinus quadratus* Saussure, 1853, extends along Pacific shorelines from Mexico to Peru (Türkay 1970).

Although both mainland populations of *Gecarcinus* have been separated by the definite closure of the Isthmus of Panama some three million years ago, the morphology of Pacific and Atlantic forms is so similar that Türkay (1970) treated *Gecarcinus quadratus* as a subspecies of *Gecarcinus lateralis*. As Türkay could not find any differences in the supplemental material he examined, he synonymized *Gecarcinus lateralis quadratus* with *Gecarcinus lateralis* (see Türkay 1973). The taxonomy of *Gecarcinus* has not been revised since Türkay's (1970, 1973) works: subsequent contributions on the ecology and general aspects of both trans-isthmian populations have followed Türkay's classification (e.g. Hartnoll 1988; Boyko 2000; Capistran-Barrados et al. 2003), while others maintained *Gecarcinus quadratus* as a valid species without further justification (e.g. Abele and Kim 1986; Tavares 1991; Ng et al. 2008).

**Figure 1.**
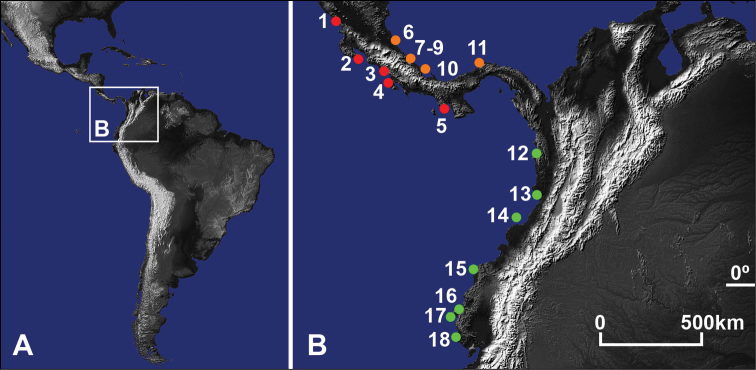
**A** Central and South America **B** Study area with locations of examined Pacific (red) and Atlantic (orange) *Gecarcinus lateralis* (Freminville, 1835) (*sensu* Türkay 1973) and *Gecarcinus nobilii* sp. n. (green). Nicaragua, Pacific coast: (**1**) Rivas, San Juan del Sur. Costa Rica, Pacific coast, Puntarenas dept.: (**2**) Pochote Beach; (**3**) Hermosa Beach; (**4**) Drake Bay. Panama, Pacific coast: (**5**) Veraguas, Santa Catalina. Costa Rica, Atlantic coast, Limón dept.: (**6**) Parismina; (**7**) Puerto Viejo; (**8**) Manzanillo; (**9**) Punta Mona. Panama, Atlantic coast: (**10**) Bocas del Toro, Bluff Beach; (**11**) Colón, Maria Chiquita. *Gecarcinus nobilii* sp. n.: Colombia, Pacific coast: (**12**) Choco, Nuquí; (**13**) Buenaventura, Chucheros Beach; (**14**) Gorgona Island. Ecuador: (**15**) Esmeraldas, Punta Galera (type location); (**16**) Manabí, Ayampe; (**17**) Plata Island; (**18**) Santa Elena.

As in trans-isthmian *Gecarcinus*, the taxonomical status of the South American Pacific population has been uncertain. Whereas Nobili (1901) described specimens from the Ecuadorian coast as *Gecarcinus ruricola*, following works do not distinguish between *Gecarcinus* from the Pacific coast of Central and South America and specimens from the latter have been referred to as *Gecarcinus quadratus* (see Rathbun 1918), *Gecarcinus lateralis quadratus* (see Türkay 1970; Prahl and Manjarres 1984) and *Gecarcinus lateralis* (see Türkay 1987). To add further complexity, Prahl and Manjarres (1984) and Türkay (1987: Fig. 7) reported white forms of *Gecarcinus* from the Pacific coast of Colombia. However, such color forms have not been observed in other areas (see Bright 1966; Chace and Hobbs 1969; Martinez and Bliss 1989), nor were such color forms observed in a field sample of 678 individuals (carapace width 20-66 mm) from the Pacific and Atlantic coastline of Central America (Table 1; Fig. 1) that was examined by one of us (RP).

**Table. 1.** Locations, date, coordinates of conducted sampling and number of examined individuals of *Gecarcinus lateralis* (Freminville, 1835) (*sensu* Türkay 1973) (carapace width 20-66 mm) (these crabs were subsequently used for analyses of stomach contents, dry weight, etc.).

Location	Date	Coordinates	N
**Pacific coast**			
Nicaragua, San Juan del Sur	15–17 July 2011	11°15’39’’N, 85°52’52’’W	54
Costa Rica, Pochote Beach	5–6 June 2011	9°44’51’’N, 85°00’01’’W	75
Costa Rica, Hermosa Beach	6–8 Nov. 2011	9°10’07’’N, 83°45’39’’W	69
Costa Rica, Drake Bay	16–18 Mar. 2011	8°41’33’’N, 83°39’42’’W	57
Panama, Santa Katalina	25–27 Oct. 2011	7°37’39’’N, 81°14’50’’W	60
**Atlantic coast**			
Costa Rica, Parismina	12–14 Nov. 2011	10°18’34’’N, 83°20’85’’W	56
Costa Rica, Puerto Viejo	26–28 May 2011	9°39’20’’N, 82°44’28’’W	68
Costa Rica, Manzanillo	20–22 Nov. 2011	9°37’53’’N, 82°39’47’’W	73
Costa Rica, Punta Mona	16–18 Nov. 2011	9°37’24’’N, 82°37’11’’W	123
Panama, Bocas del Toro, Bluff beach	10–12 Oct. 2011	9°23’25’’N, 82°14’14’’W	23
Panama, Maria Chiquita	20–22 Oct. 2011	9°26’42’’N, 79°45’44’’W	19

An Internet search revealed additional photographs of *Gecarcinus* individuals from the Pacific coast of South America with a body color different from the specimens that were collected on the Central American coasts. Further examination of museum specimens and re-examination of the freshly collected specimens from Central America has revealed that the color differences are accompanied by differences in morphological structures as well. In accordance with these differences, a new species of *Gecarcinus* is herein proposed.

Specimens from the following institutions were examined: Academy of Natural Sciences of Drexel University, Philadelphia, USA (ANSP); Natural History Museum of Los Angeles County, Los Angeles, USA (LACM); Muséum National d’Histoire Naturelle, Paris, France (MNHN); Museo de Zoología, Universidad de Costa Rica, San José, Costa Rica (MZUCR); Naturhistorisches Museum, Basel, Switzerland (NHMB) and National Museum of Natural History, Smithsonian Institution, Washington, D.C., USA (USNM).

## Taxonomy

### Gecarcinus

Taxon classificationAnimaliaDecapodaGecarcinidae

Leach, 1814

Gecarcinus Leach, 1814: 430.

#### Type species.

*Cancer ruricola* Linnaeus, 1758 (original combination).

#### Diagnosis.

Exopod not projecting beyond third maxilliped ischium-merus articulation, without flagellum; palp concealed by third maxilliped merus margin. Mesial segment of first male gonopod distally reduced and terminal segment basally exposed, terminal segment projecting beyond apical setae.

#### Remarks.

Because further evidence challenging Türkay’s (1973) synonymization of *Gecarcinus quadratus* with *Gecarcinus lateralis* has not been presented to date, we follow his taxonomy and treat *Gecarcinus quadratus*, described from the Pacific mainland, as a junior synonym of *Gecarcinus lateralis*.

#### Key to the species of *Gecarcinus* Leach, 1814

**Table:** 

1	Mesial lobe of infraorbital margin curved around ventrolateral edge of carapace front. Third maxilliped merus covers epistome and can reach carapace front. Spines on lateral carina of dactylus and carpus of ambulatory legs prominently developed in adults	*Gecarcinus ruricola* (Linnaeus)
–	Contact between carapace front and mesial lobe of infraorbital margin straight (Fig. 2B, C). Third maxilliped merus at the farthest reaching epistome. Spines on lateral carina of dactylus and carpus of ambulatory legs weakly developed to absent	2
2	Carapace front wider than distance between mesial ends of suborbital cristae (Figs 2C; 3B). Width of mesial lobe of infraorbital margin at point of contact with carapace front longer than shortest distance between carapace front and mesial end of suborbital crista (Figs 2C; 3B). Light lateral margin on dorsal carapace without lighter anterolateral and posterior patches (Figs 4C; 5D)	*Gecarcinus nobilii* sp. n.
–	Carapace front about as wide as distance between mesial ends of suborbital cristae (Figs 2B; 6B, E). Width of mesial lobe of infraorbital margin at point of contact with carapace front shorter than shortest distance between carapace front and mesial end of suborbital crista (Figs 2B; 6B, E). Without light lateral margin on dorsal carapace (Pacific, Fig. 5A; Atlantic, Fig. 5C) or with light lateral margin with anterior and posterior yellow to orange patches (Atlantic, Fig. 5B)	*Gecarcinus lateralis* (Fréminville)

**Figure 2.**
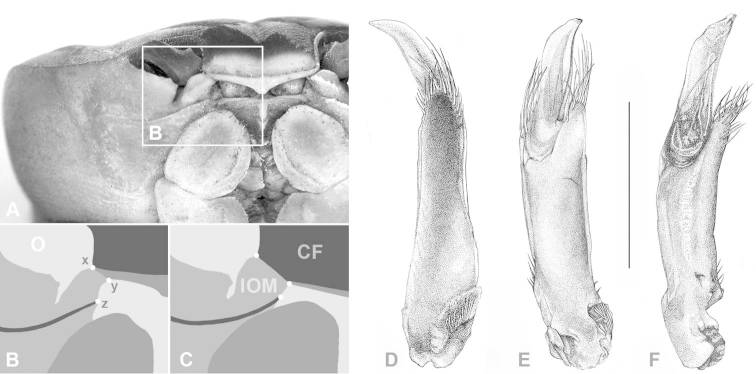
(**CF**) Carapace front; (**O**) orbit; (**IOM**) mesial lobe of infraorbital margin; (**x**) widest width of CF; (**z**) mesial end of suborbital crista; (**x–y**) width of IOM at point of contact with CF; (**y–z**) shortest distance between CF and mesial end of suborbital crista; **A**, **B** Atlantic *Gecarcinus lateralis* (Freminville 1835), male, carapace width (CW) 31 mm, Costa Rica, Puerto Viejo **C** *Gecarcinus nobilii* sp. n., holotype, male, CW 31 mm, Ecuador, Punta Galera (LACM CR 1968-477). First male gonopod: *Gecarcinus nobilii* sp. n., holotype: **D** mesial view **E** lateral view **F** Pacific *Gecarcinus lateralis* (*sensu* Türkay 1973), CW 31 mm, Costa Rica, Hermosa Beach, lateral view; Scale bar = 5 mm.

**Figure 3.**
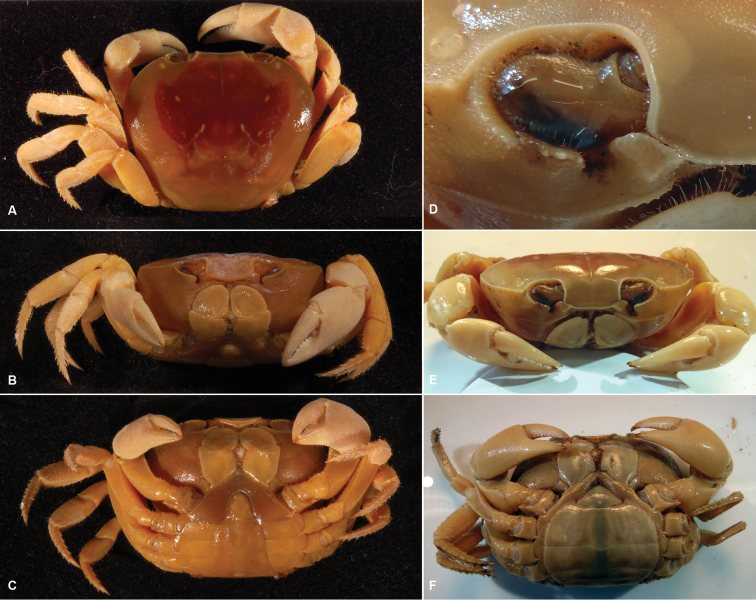
*Gecarcinus nobilii* sp. n., holotype, male, carapace width 31 mm, Ecuador, Punta Galera (LACM CR 1968-477), preserved in alcohol (color faded): **A** dorsal view **B** frontal view **C** ventral view. Paratype, female, Ecuador, St. Elena (MNHN, B12314), preserved in alcohol (color faded): **D** carapace front **E** frontal view **F** ventral view.

**Figure 4.**
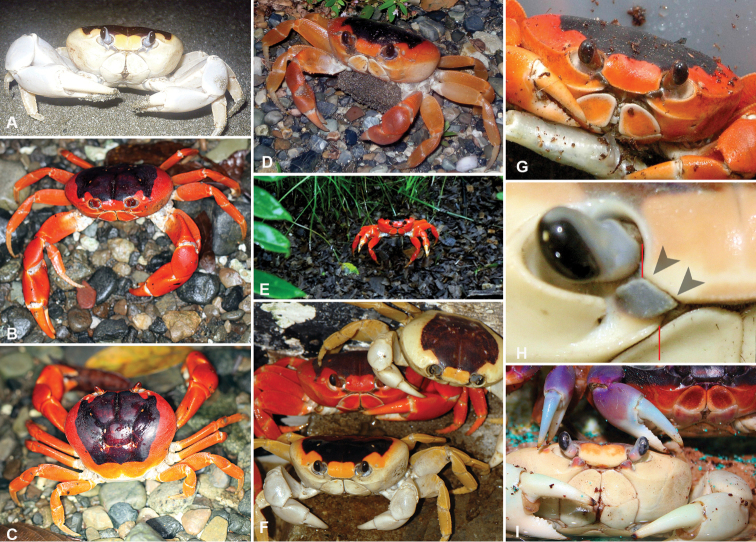
*Gecarcinus nobilii* sp. n., color in life; Colombia: Gorgona Island **A** male, photo by Karla Garcia Burneo (Peru) **B**, **C** male, photos by Rhett A. Butler (USA) **D** female, Buenaventura dept., Chucheros Beach, photo by Elena Gómez **E** sex unknown, Chocó dept., Nuquí prov., Canangucho Forest Reserve. Ecuador: **F** sex unknown, Manabí prov., Ayampe, photo by David Liebman (USA). Captive individuals from the pet trade, origin unknown: **G** sex unknown, photo by Oliver Mengedoht (Germany) **H**, **I** females, photos by John Beatty (USA) (the individual shown above in Fig. 1 belongs to the Pacific population of *Gecarcinus lateralis* (sensu Türkay 1973), please note the differences in the mesial lobe of the infraorbital margin).

**Figure 5.**
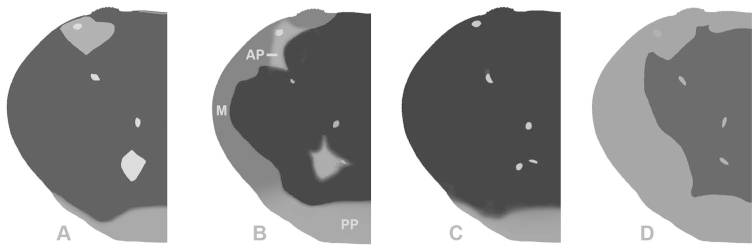
Dorsal carapace pattern (shape of light patches could vary slightly): *Gecarcinus lateralis* (Freminville 1835) (*sensu* Türkay 1973): **A** Pacific coast of Central America. Atlantic coast of Central America: **B** form with lateral margin (**M**) on dorsal carapace and orange patches at anterolateral (**AP**) and posterior (**PP**) carapace border; **C** form without lateral margin on dorsal carapace **D** *Gecarcinus nobilii* sp. n.

**Figure 6.**
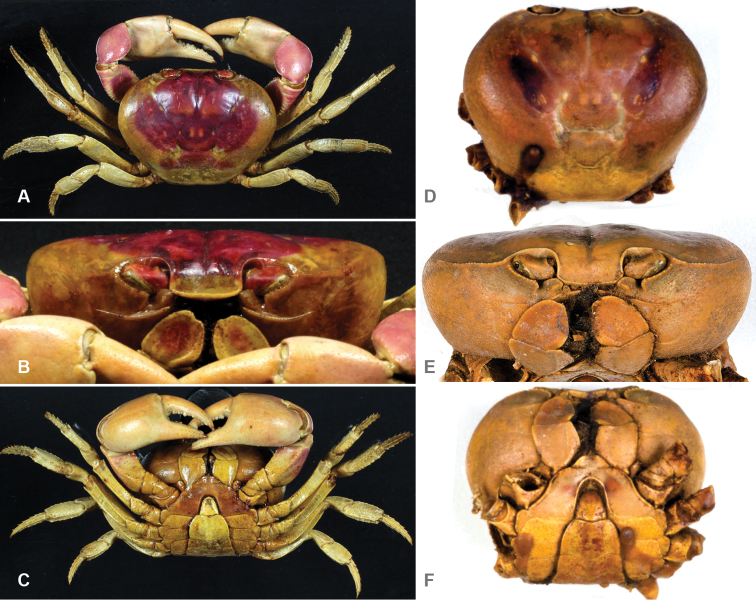
**A–C** lectotype of *Gecarcinus lateralis* (Freminville, 1835), male, carapace width 47.2 mm, Guadeloupe (MNHN-3758) (dried specimen, color faded) **D–F** syntype of *Gecarcinus quadratus* Saussure, 1853, male, carapace width 39.7 mm, Mexico, Mazatlan (ANSP-CA3741) (dried specimen, color faded) (photos by Paul Callomon, Academy of Natural Sciences of Drexel University, Philadelphia).

### Gecarcinus nobilii sp. n.

Taxon classificationAnimaliaDecapodaGecarcinidae

http://zoobank.org/F3BA2617-49DF-4C26-ACB5-5407122301FF

Figs 2C–E ; 3 ; 4 ; 5D

Gecarcinus ruricola . – Nobili 1901: 46.
Gecarcinus (Gecarcinus) lateralis quadratus . – Türkay 1970: 338. – Prahl and Manjarres 1984: 155.
Gecarcinus lateralis . – Türkay 1987: 147, fig. 7.

#### Material examined.

Holotype: male, carapace width (CW) 31 mm, Ecuador, Punta Galera, 0°50'N, 80°6'W, shore, collected near a pile of fairly fresh cow manure, Te Vega Expeditions, Sta. NO. XVIII-6, 22 April 1968. Coll. E. Ball (LACM CR 1968-477). Paratypes: 1 male, CW 26 mm, same location data as holotype (LACM CR 1968-478); 1 female, Ecuador, St. Elena (MNHN- B12314); 3 females, CW 28, 23, 23.5 mm, Ecuador, Esmeraldas (NHMB-NMB1010b). (Note: The female paratype (MNHN-B12314) (Fig. 3D–F) of *Gecarcinus nobilii* sp. n. was labeled as "Gecarcinus festae Nobili/(co-type)/St. Helena/Festa/Museum Paris/Ecuador/Nobili 1901” without information about the label author. Nobili never published a description of a species by this name. In 1901 he described *Sesarma festae* and *Uca festae* and in the same contribution referred to specimens of *Gecarcinus* collected in Ecuador as *Gecarcinus ruricola*, which is restricted to West Atlantic Islands (Türkay 1970; Bright and Hogue 1972).

Additionally, high resolution photographs of 14 captive individuals with unknown origin and 17 individuals taken at the following locations (confirmed by the photographers): Gorgona Island (Colombia) (Fig. 4A–C), Chucheros Beach (Buenaventura, Colombia) (Fig. 4D), Canangucho Forest Reserve (Nuquí, Chocó, Colombia) (Fig. 4E), Ayampe (Manabí, Ecuador) (Fig. 4F), and Isla de la Plata (Ecuador), were examined.

#### Comparative material.

*Gecarcinus lateralis* (Freminville, 1835): lectotype male, CW 47.2 mm, Guadeloupe, M. Beaupertius leg. (MNHN-3758). Paralectotypes: 1 male, CW 50 mm, Guadeloupe, M. Beaupertius leg. (MNHN-3757); 1 female, CW 28 mm, same data as preceding specimen. 1 male, CW 32 mm, Martinique, M. Bellanger leg. (MNHN-3756); 1 female, CW 37 mm, Martinique, Bellanger leg., 24.09.1964 (MNHN-3755). *Gecarcinus quadratus* Saussure, 1853: syntype male, CW 39.7 mm, Mexico, Mazatlan (ANSP-CA3741). Pacific *Gecarcinus lateralis* (*sensu* Türkay 1973): 1 male, Mexico, Sinaloa, Estero el Verde (MNHN-B20900). 1 male, Costa Rica, Guanacaste, Playa del Coco, 5.8.1967, W. McCaul leg. (MZUCR13-01). 1 male, 1 female, Costa Rica, Puntarenas, Parque Nacional Manuel Antonio, 4.2.1995, J. Cortés leg. (MZUCR-2016). 1 male, Panama, Canas Island, Los Santos, Turtle Hatchery on S Beach, J. Frazier leg. (USNM-190711). Additional comparative material is cited in Table 1.

#### Derivation of specific epithet.

The species is named in honor of Giuseppe Nobili, who provided important contributions on the knowledge of crustaceans and built the crustacean collection in the Turin Museum (Italy). The species name is a noun in the genitive case.

#### Diagnosis.

Frontal width distinctly wider than the distance between the mesial ends of the suborbital cristae (Figs 2C; 3B, E). Width of mesial lobe of infraorbital margin at point of contact with carapace front longer than shortest distance between carapace front and mesial end of suborbital crista (Figs 2C; 3B, E). Light lateral margin on dorsal carapace without lighter anterolateral and posterior patches (Figs 4C; 5D); cheliped carpus and palm homogeneously red or white (Fig. 4).

#### Description.

Carapace transversely ovate, widest in anterior half, dorsal surface smooth. Cardiac, gastric and branchial chambers moderately swollen (Fig. 3B, E). Median groove distinct, cervical and urogastric grooves weakly developed; three relatively small pits anterior (close to orbit), median and posterior of cervical groove, one in urogastric groove (Fig. 3A). Supraorbital margin gently sinuous, with small granules, confluent with anterolateral margin; exorbital tooth weakly developed, tip not over-reaching orbit (Fig. 3B, D, E); granules along anterolateral and orbital margins weakly developed. Eyes well developed, filling orbital cavity; eyestalks short (Fig. 3B, E). Carapace front distinctly wider than the distance between mesial ends of the suborbital cristae (Figs 2C; 3B, E), deflexed downwards, concealing basal segments of antennules. Width of mesial lobe of infraorbital margin at point of contact with carapace front longer than shortest distance between carapace front and mesial end of suborbital crista (Figs 2C; 3B, D; 4H). Suborbital, pterygostomial regions sparsely granular laterally. Subhepatic region with rounded postero-lateral margins, with rows of small granules. Epistom linear, sunken.

Third maxilliped merus and outer ventral orbital border furnished with long setae (Fig. 3D); third maxilliped merus enlarged, reaching mesial border of suborbital crista, triangular, apex straight or moderately convex (Figs 3B, 4I); exopodit short, not protruding beyond third maxilliped ischium-merus joint, without flagellum; palpus concealed by external border of third maxilliped merus.

Chelipeds subequal; in large males larger with respect to the carapace width, surfaces relatively smooth, weakly granulate. Merus with transversal rows of small tubercles; dorsal margin rugose or with moderately developed, obtuse granules; ventral margin lined with weakly developed granules, otherwise smooth. Carpus with 2–5 well developed triangular inner subdistal tooth (Fig. 3A, B, E). Merus and carpus margins smooth in large individuals. Chela large, length not exceeding carapace width, surfaces smooth; lower margin gently sinuous. Fingers slightly shorter or as long as palm, tapering, gently curved, drop-shaped in cross-section, proximal half with irregular arranged pores and low, pectinated teeth; teeth on distal portion of finger arranged on well defined, subparallel longitudinal ridges, longitudinally separated by grooves with pores. Cutting margins with distinct triangular teeth along length; fingers without or with small gap between them when closed, ending in sharp, pectinated tips.

Ambulatory legs with second pair longest, last pair shortest; surfaces smooth to slightly rugose. Merus dorso-laterally flattened, cross-section triangular, stout; with transversal rows of small tubercles, dorsal margin distinct, granulated, with separate, short setae. Carpus stout, subtriangular in cross-section; dorsal surface with three carinae, median carina distinct, serrated or granular; dorso-lateral carinae weakly developed or absent; margins and carinae lined with separate, short setae. Propodus subrectangular in cross-section; lateral margins subparallel, lined with low, obtuse spines and separate, short setae (Fig. 3A–C). Dactylus elongate, styliform, gently curving, subquadrate in cross-section, margins lined with distinct spines and separate, short setae; apical half of spines and dactylus tip corneous; lateral carina of dactylus weakly developed or absent (Fig. 3A–C).

Male abdomen relatively broad (Fig. 3C), all abdominal somites and telson distinct, freely articulating. Somite 1 filling space between last pair of ambulatory legs, longitudinally very narrow. Shape of somite 2 similar to somite 1 but narrower. Somites 3–5 increasingly trapezoidal in shape, lateral margins relatively straight. Somite 6 longest, longer than telson, distinctly wider than long, with lateral margin strongly convex. Telson sub-triangular, narrowest abdominal segment; as long as wide, lateral margins gently concave to almost straight, tip rounded (Fig. 3C).

First male gonopod with basal and terminal segment (Fig. 2E). Basal segment stout, straight, with digiform projection on distomesial end, projection directed in same manner with distal segment, fringed with long setae. Terminal segment about one-third of first gonopod (when seen from lateral view, Fig. 2E), folded longitudinally, compressed dorsoventrally, tapering and curved distally, slightly projecting over distal setae, laterally with narrow, longitudinal furrow.

Sex independent color dimorphism: red and white males and females (Fig. 4). Both forms with dark median pattern and contrasting light lateral margin on dorsal carapace without lighter anterior and posterior patches (Fig. 5D), margin of same color as lateral carapace; dark dorsal carapace pattern with pointed anterolateral edges anteriorly reaching the orbits (Fig. 5D). Mesial lobe of infraorbital margin mostly grey (Fig. 4). Red form with red lateral margin on dorsal carapace. Carapace pits white to orange. Legs and chelipeds uniformly red, inner sides of fingers cream to white, margin of the third maxilliped merus, coxa, basis and ischium of chelipeds and ambulatory legs whitish. In white form, lateral margin on dorsal carapace orange/white or completely white. Ventral carapace and chelipeds white, legs and carapace pits light orange to white (Fig. 4).

#### Geographic distribution.

*Gecarcinus nobilii* sp. n. is currently known to occur from Punta Galera and St. Elena (Ecuador). It is also documented in photographs taken at Gorgona Island (Colombia) (Fig. 4A–C), Chucheros Beach (Buenaventura, Colombia) (Fig. 4D), Canangucho Forest Reserve (Nuquí, Chocó, Colombia) (Fig. 4E), Ayampe (Manabí, Ecuador) (Fig. 4F), and Isla de la Plata (Ecuador). Individuals of *Gecarcinus* previously reported from Peru (Türkay 1970) may also refer to *Gecarcinus nobilii* sp. n.

Available data and the photographs found during the Internet search suggest that *Gecarcinus nobilii* sp. n. replaces Pacific *Gecarcinus lateralis* between the Darien province (Panama) and the Choco dept. (Colombia). In addition to the individuals of Pacific *Gecarcinus lateralis* found during fieldwork (Table 1), the Internet search revealed numerous photographs of Pacific *Gecarcinus lateralis* from Central America. However, there are no photographs of Pacific *Gecarcinus lateralis* from within the distributional area of *Gecarcinus nobilii* sp. n.

#### Remarks.

**Figure 7.**
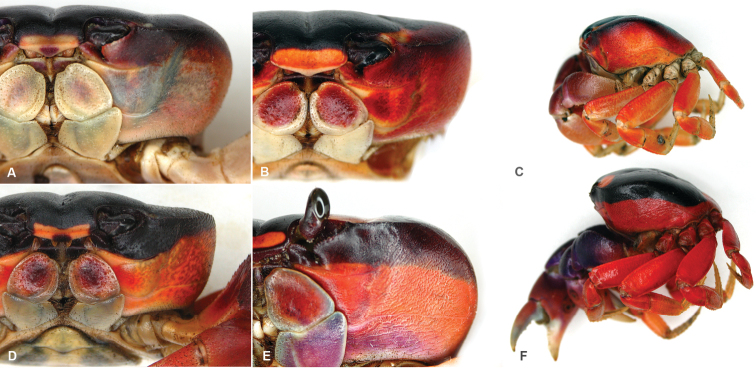
*Gecarcinus lateralis* (Freminville, 1835) (*sensu* Türkay 1973), frontal and lateral views, color in life, in hard-shell condition. Atlantic coast: Costa Rica, Puerto Viejo: **A** male, carapace width (CW) 44 mm **B**, **C** male, CW 29 mm **D** female with contrasting dorso- and ventrolateral color, CW 32 mm. Pacific coast: Costa Rica, Playa Hermosa: **E** male, CW 58 mm **F** male, CW 38 mm.

**Figure 8.**
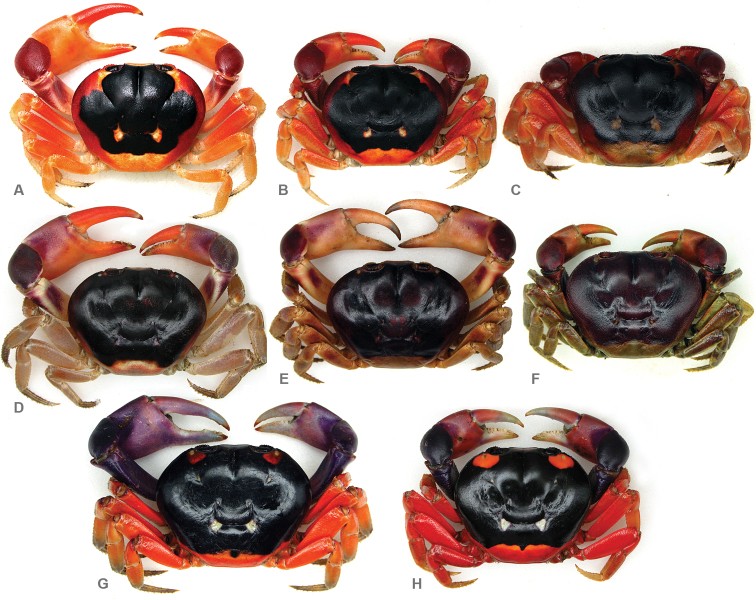
*Gecarcinus lateralis* (Freminville, 1835) (*sensu* Türkay 1973), dorsal view, color in life, in hard-shell condition. Atlantic coast, Costa Rica, Puerto Viejo, showing the forms limiting the range of color variability: **A** male, carapace width (CW) 39 mm **B** male, CW 29 mm **C** female, CW 32 mm **D** male, CW 44 mm **E** male, CW 47 mm **F** female, CW 33 mm. Pacific coast: Costa Rica, Playa Hermosa: **G** male, CW 58 mm **H** male, CW 38 mm.

The resemblance of the general habitus, the shape and the surface structure of carapace, chelipeds, ambulatory legs (Figs 3; 6–8) and the first male gonopod (Fig. 2E, F) indicate a close relationship between all mainland *Gecarcinus* populations. However, the trans-isthmian populations of *Gecarcinus lateralis* differ from *Gecarcinus nobilii* sp. n. by having a carapace front approximately as wide as the distance between the mesial ends of the suborbital cristae, and the width of the mesial lobe of the infraorbital margin at the point of contact with the carapace front is shorter than the shortest distance between the carapace front and the mesial end of the suborbital crista (Figs 2B; 6B, E; 7A, B).

A character commonly used to distinguish between species of the Gecarcinidae is the shape of the third maxilliped merus margin (e.g. Rathbun 1918; Türkay 1970; Perger et al. 2011). According to Rathbun (1918), the Atlantic population of *Gecarcinus lateralis* has an emarginated margin and the Pacific population a continuous margin. However, Bott (1955) and Türkay (1970, 1973) recognized the presence of both character states in trans-isthmian populations from Central America, South America and the Antilles, which we also observed in our sample from Central America. In contrast, all examined specimens of *Gecarcinus nobilii* sp. n. have a relatively continuous third maxilliped margin.

In addition to observed differences in morphological structures, the body color of Pacific *Gecarcinus lateralis* (*sensu* Türkay 1973) differs from *Gecarcinus nobilii* sp. n. in the following manner: Dorsal carapace without light lateral margin (Fig. 5A), dark dorsal carapace pattern extending laterally over anterolateral carapace border (Fig. 7E, F); orange patch at anterolateral and posterior carapace border (Figs 5A; 8G, H). Cheliped carpus and palm violet, rarely purplish (Fig. 8G, H). Atlantic *Gecarcinus lateralis* are distinguished from *Gecarcinus nobilii* sp. n. by following color differences: Light lateral margin on dorsal carapace with lighter (yellow to orange) patch at anterolateral and posterior carapace border (Figs 5B; 8A, B) or margin and anterolateral patch absent (Figs 5C; 8D–F). Ventrolateral carapace color heterogeneous (Fig. 7A–C). Cheliped carpus and palm heterogeneously purple, red, orange and/or whitish (Fig. 8A–F).

Apart from the consistent color differences between *Gecarcinus nobilii* sp. n. and the other mainland populations of *Gecarcinus*, which provided the starting point for this contribution, the color differences between the trans-isthmian populations of *Gecarcinus* (Table 2; Figs 5A–C; 7; 8) also drew our attention. According to Bright (1966), *Gecarcinus* from the Pacific coast of Central America has a brownish-red carapace and chelipeds with a purple tinge, while *Gecarcinus lateralis* from the Atlantic coast of Central America has a dark red carapace pattern and reddish-gray chelipeds. Chace and Hobbs (1969) provided the first color description of *Gecarcinus lateralis* from the West Indies, which widely agreed with the brief description of the *Gecarcinus* specimens from the Pacific coast of Central America by Bright (1966). Martinez and Bliss (1989) later described and illustrated the color of *Gecarcinus lateralis* from Bermuda and Bimini Islands in detail. The authors observed a remarkable variability in color and even color changes in single individuals, leaving open the possibility that Pacific *Gecarcinus lateralis* might also approach the range of variability observed in Atlantic *Gecarcinus lateralis*.

**Table 2.** Comparison of color in life for mainland *Gecarcinus* spp. (carapace width of *Gecarcinus lateralis* (Freminville, 1835) (*sensu* Türkay 1973) 20-66 mm); (IOM) mesial lobe of infraorbital margin; (SUB) suborbital crista; (ACB) anterolateral carapace border.

	Atlantic *Gecarcinus lateralis* (n = 362)	Pacific *Gecarcinus lateralis* (n = 316)	*Gecarcinus nobilii* sp. n. (n = 19)
**Color polymorphism**	Morphs with transitional forms	-	Red and white forms
**Light lateral margin on dorsal carapace**	Present or absent, when absent, dark median pattern laterally extending over ACB with smooth transition to color of ventrolateral carapace	Absent, dark median pattern laterally extending over ACB, anteriorly mostly reaching SUB, with sharp contrast to color of ventrolateral carapace	Present, contrasting to dark median pattern, same color as lateral carapace, in some white forms with orange tinge
**Orange patch at anterolateral carapace border**	In forms with lateral margin on dorsal carapace, patch with smooth transition to bordering color, only posteriorly bordered by dark carapace pattern; patch absent in forms without light lateral margin on carapace	Always present, encircled by dark, sharply contrasting dorsal carapace color	Absent
**Patch at posterior lateral urogastric groove**	In forms with lateral margin on dorsal carapace heterogeneously yellowish, grayish, cream, sometimes with smooth transition to lateral margin;<br/> weakly developed to absent in forms without lateral margin	Homogeneously whitish, subtriangular to rhomboid, well defined, sharply contrasting with surrounding dark carapace color	Absent
**Ventro-lateral carapace color**	Heterogeneous, transition between dorsal and ventral carapace color	Uniformly bright deep orange to red, sharply contrasting to dorsolateral carapace color	Homogeneously red or white
**Cheliped palm color**	Orange to red, often with purple tinge anteriorly, or orange-purplish-whitish	Deep violet, sometimes with purplish tinge	Uniformly red or white
**Cheliped dactylus color**	Light orange, orange to light red, cutting edges cream	Completely white or base light violet, purplish, becoming white distally	Red or white, cutting edges cream or white
**IOM color**	As suborbital area		Mostly grey

While the color of examined *Gecarcinus lateralis* from the Atlantic coast (n= 362) varied within the range described by Martinez and Bliss (1989) (see Fig. 8A–F), the color of individuals of Pacific *Gecarcinus lateralis* examined in this study (n= 316) showed little variation (Fig. 8G, H) and did not approach the range of variability found in *Gecarcinus lateralis* from the Atlantic coast of Central America (Table 2) and Bermuda and Bimini Islands (see Martinez and Bliss 1989). In a sample totaling 678 individuals, only a single female from the Atlantic coast (Fig. 7D) did not clearly match with each of the color characters attributed to either the Atlantic and Pacific population (Table 2). This individual had a dark dorsal carapace color expanding laterally over the dorsolateral carapace border and sharply contrasting with the ventrolateral carapace color. However, the remaining characters (Table 2) agreed with the other individuals of Atlantic *Gecarcinus lateralis*. Within several groups of decapod crustaceans, color and color pattern reliably distinguish a number of species that differ little in morphology (e.g. Bruce 1975; Knowlton 1986). Color pattern-level and genetic differentiation between cryptic species has been observed in hermit crabs (e.g. Malay and Paulay 2009), spiny lobsters (Ravago and Juinio-Menez 2003), porcelain crabs (Hiller et al. 2006) and in the Gecarcinidae genus *Discoplax* A. Milne-Edwards, 1867 (Ng and Davie 2012).

Studies of genetic divergence and reproductive isolation are needed to evaluate whether *Gecarcinus quadratus* should be retained as a synonym of *Gecarcinus lateralis*, or alternatively, the trans-isthmian populations of *Gecarcinus lateralis* represent allopatric sister species.

#### Evolutionary relationships.

When we consider the closure of the Panamanian Isthmus as a calibration point for morphological divergence between the trans-isthmian populations of *Gecarcinus lateralis*, the virtual lack of morphological differentiation (other than color) between them and the distinctness of *Gecarcinus nobilii* sp. n. suggests that *Gecarcinus nobilii* sp. n. evolved from a common ancestor before the Isthmus closed. The common ancestor of the trans-isthmian *Gecarcinus lateralis* may have been restricted to North America and/or the emerging Isthmus, which is assumed to have been a peninsula of North America (Kirby et al. 2008), and the ancestor of *Gecarcinus nobilii* sp. n. to South America. Nevertheless, the distribution of the gecarcinid *Johngarthia planata* Stimpson, 1860, from Gorgona Island to Mexico (reviewed by Perger et al. 2013) and *Cardisoma crassum* Smith, 1870, from Peru to Mexico (Türkay 1970) as well as the absence of *Gecarcinus nobilii* sp. n. from the Atlantic coast of South America suggests a more complex pattern. A promising approach to further investigation of the speciation processes in Neotropical Gecarcinidae may be the evaluation of how the connection between the habitats of the adults via sea currents may have changed during the formation of the Isthmus (see Schneider and Schmittner 2006; Molnar 2008). Further studies should also take into account that even today, as indicated by the actual distribution, there appear to be mechanisms separating the *Gecarcinus* populations from the Central and South American Pacific coast.

## Supplementary Material

XML Treatment for Gecarcinus

XML Treatment for Gecarcinus nobilii
